# Efficient Cross-Correlation Filtering of One- and Two-Color Single Molecule Localization Microscopy Data

**DOI:** 10.3389/fbinf.2021.739769

**Published:** 2021-11-04

**Authors:** Angel Mancebo, Dushyant Mehra, Chiranjib Banerjee, Do-Hyung Kim, Elias M. Puchner

**Affiliations:** ^1^ School of Physics and Astronomy, University of Minnesota, Minneapolis, MN, United States; ^2^ Department of Physiology and Biomedical Engineering, Mayo Clinic, Rochester, MN, United States; ^3^ Department of Biochemistry, Molecular Biology, and Biophysics, University of Minnesota, Minneapolis, MN, United States

**Keywords:** single-molecule localization microscopy, photo-activated localization microscopy, cross-correlation, colocalization, clustering

## Abstract

Single molecule localization microscopy has become a prominent technique to quantitatively study biological processes below the optical diffraction limit. By fitting the intensity profile of single sparsely activated fluorophores, which are often attached to a specific biomolecule within a cell, the locations of all imaged fluorophores are obtained with ∼20 nm resolution in the form of a coordinate table. While rendered super-resolution images reveal structural features of intracellular structures below the optical diffraction limit, the ability to further analyze the molecular coordinates presents opportunities to gain additional quantitative insights into the spatial distribution of a biomolecule of interest. For instance, pair-correlation or radial distribution functions are employed as a measure of clustering, and cross-correlation analysis reveals the colocalization of two biomolecules in two-color SMLM data. Here, we present an efficient filtering method for SMLM data sets based on pair- or cross-correlation to isolate localizations that are clustered or appear in proximity to a second set of localizations in two-color SMLM data. In this way, clustered or colocalized localizations can be separately rendered and analyzed to compare other molecular properties to the remaining localizations, such as their oligomeric state or mobility in live cell experiments. Current matrix-based cross-correlation analyses of large data sets quickly reach the limitations of computer memory due to the space complexity of constructing the distance matrices. Our approach leverages k-dimensional trees to efficiently perform range searches, which dramatically reduces memory needs and the time for the analysis. We demonstrate the versatile applications of this method with simulated data sets as well as examples of two-color SMLM data. The provided MATLAB code and its description can be integrated into existing localization analysis packages and provides a useful resource to analyze SMLM data with new detail.

## Introduction

The subcellular localization of proteins and their interaction with other biomolecules is a critical determinant of their function. For instance, proteins have been shown to form clusters in nuclear condensates which affect chromatin organization and gene regulation (Cisse et al., 2013; Larson et al., 2017; Cho et al., 2018; Chong et al., 2018; Sabari et al., 2018; Cai et al., 2019; McSwiggen et al., 2019). Cell surface receptors such as TNFα, EGFR, and TLR4 have been shown to form functional clusters in the cell membrane that enhance signaling (Krüger et al., 2017, 4; van Lengerich et al., 2017; Karathanasis et al., 2020). In the immunological synapse, various receptors and signaling proteins are co-clustered or excluded in supramolecular activation clusters (Hartman et al., 2009; Pageon et al., 2016b). In most contexts, the fraction of the proteins that cluster or co-cluster with other proteins have different biophysical and biochemical properties compared to the non-clustered ones.

Fluorescence microscopy has become a prominent technique to study the sub-cellular distribution and colocalization of specifically labeled proteins in cells. However, many intracellular structures and proteins clusters are too small or too closely spaced to be resolved below the optical diffraction limit of conventional fluorescence microscopy. Super-resolution microscopy techniques such as single molecule localization microscopy (SMLM) (Betzig et al., 2006; Rust et al., 2006) overcome these challenges. Instead of imaging all fluorophores at the same time, SMLM employs fluorophores that are predominantly in a dark state but switch to a fluorescent state either intrinsically or induced by irradiation of a certain wavelength of light (Patterson and Lippincott-Schwartz, 2002; Betzig et al., 2006; Rust et al., 2006). In this way, only sparse and spatially well separated single molecules are in a fluorescent state and are detected in a single imaging frame. By recording many frames, all individual fluorophores are then imaged over time. A super-resolution image is then constructed by fitting all sparse fluorophores in each frame with a point-spread function (PSF) or Gaussian and by superimposing the center coordinates of all localizations that typically have a precision of ∼20 nm.

In contrast to pixel-intensity information of conventional fluorescence microscopy, SMLM data is based on coordinates, widths, heights etc., of all fitted single fluorophores and therefore presents unique opportunities for secondary data analysis. For instance blink-correction algorithms have been developed to correct repeated localizations of the same fluorophore that arise from the complicated photophysics (Lee et al., 2012; Rollins et al., 2015; Hummer et al., 2016; De Zitter et al., 2020) and to count the number of molecules on an organelle or cluster (Puchner et al., 2013). Various clustering algorithms have been developed to quantify the degree or variability of clustering of a protein of interest under various conditions. Examples include local clustering algorithms that define boundaries of dense localizations (Ester et al., 1996; Perry, 2004; Owen et al., 2010; Pageon et al., 2016a; Griffié et al., 2016; Levet et al., 2019; Khater et al., 2020; Nino et al., 2020; Simoncelli et al., 2020; Williamson et al., 2020; Marenda et al., 2021; Nieves et al., 2021) or bulk metrics based on the radial distribution or pair-correlation function that quantify the density of localization pairs as a function of their distance to each other (Ripley, 1979; Kiskowski et al., 2009; Sengupta et al., 2011; Veatch et al., 2012; Stone and Veatch, 2015). Importantly, these analysis methods can be expanded to two-color SMLM data to quantify the colocalization and structural relation of two proteins. For instance, cross-correlation and pair correlation analysis has been used to study co-localization among synaptic membrane receptors (Malkusch et al., 2012; Pageon et al., 2016a, 2016b; Krüger et al., 2017; Khater et al., 2018, 2019; Lagache et al., 2018; Kennedy et al., 2019; Karathanasis et al., 2020; Simoncelli et al., 2020) and quantify the density of accessible DNA domains colocalized with nuclear condensates and other nuclear landmarks (Lee, 2019; Xie et al., 2020).

For the analysis of any SMLM data set that exhibits clustering or colocalization of two different proteins, it would be desirable to separate the molecule list based on the proximity of proteins to each other. In this way localizations from clustered or colocalized proteins can be separately visualized and analyzed to study how their properties such as molecule number or their structure differs from the rest of localizations that are not clustered or colocalized. While pair-or cross-correlation analysis in principle allows to make this separation based on a distance threshold, the calculation of the distance matrix is memory intensive and can’t be use over entire field of view of a typical mammalian cell due to the large number of N localizations. Both the memory requirement and calculation time scales as N^2^. An approach to generate a cross correlation curve is to use small sections of data (Kennedy et al., 2019), however, most of the localizations are discarded. Fourier transformations are also used to calculate the cross-correlation curve across the full field of view (Kiskowski et al., 2009; Liu et al., 2014, 2; Xie et al., 2020) but these methods discard localizations that generate pairwise distances during analysis. Therefore, localizations that are within specific distances or are colocalized with a protein of interest cannot be separated and further analyzed. These existing methods provide a coarse-grained representation of the cross-correlation curve and may miss relevant transitions occurring at smaller differences in distances that could provide insight into protein oligomerization.

**FIGURE 1:**
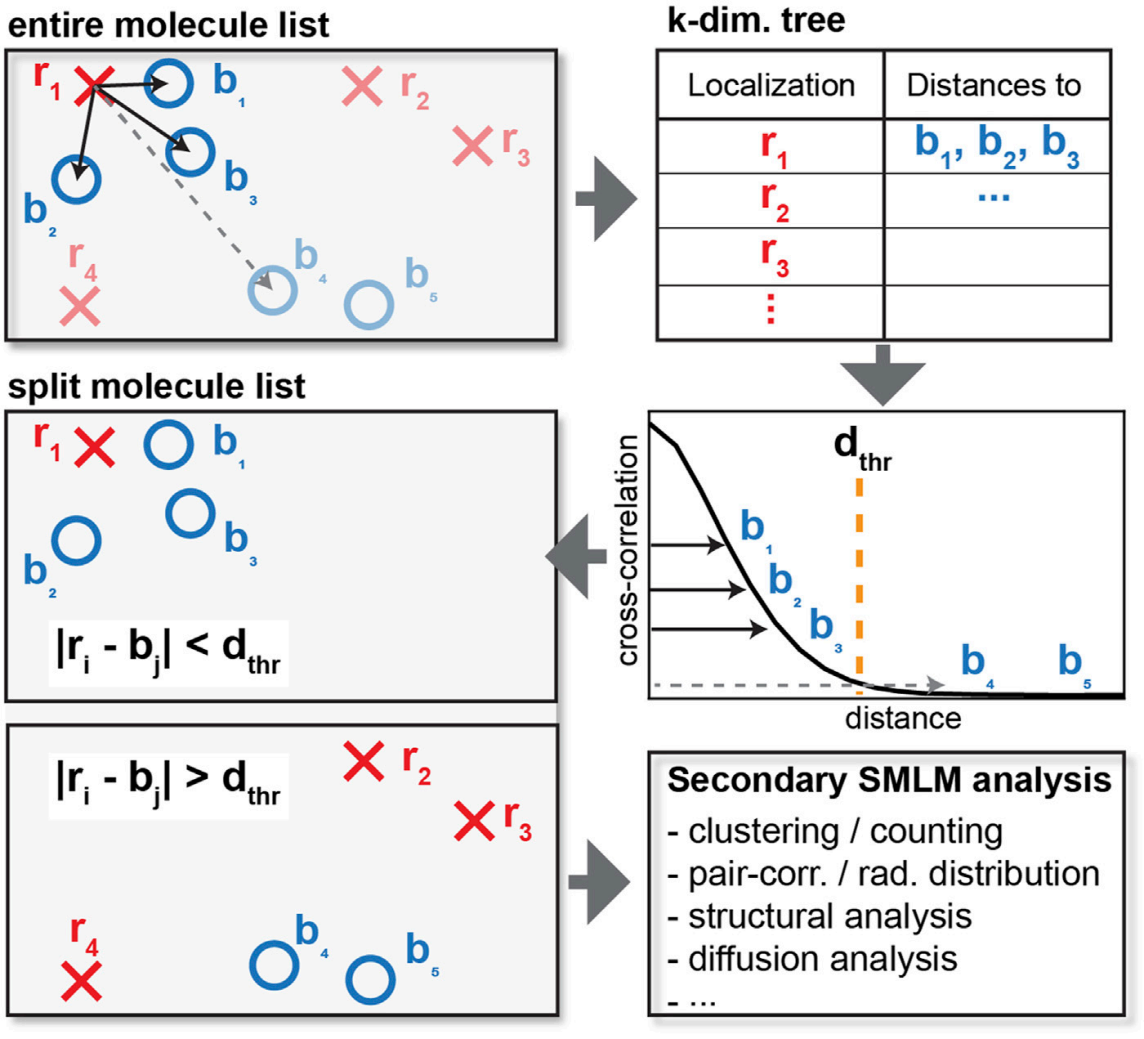
Schematics of cross-correlation. For each localization of one type (red crosses), the distance to each of the other type (blue circles) is computed and tabulated. Instead of calculating the distance of every pair of red crosses and blue circles, a distance cutoff is applied to only consider interparticle distances within relevant length scales. When the distance exceeds the cutoff as shown by the dashed line, the distance is not tabulated, resulting in reduced memory requirements. The list of localization can then be split into localization that do and do not colocalize or appear within the specified distance cutoff for further downstream analysis.

Here, we address these limitations by developing a memory efficient approach using k-dimensional trees (Bentley, 1975) to efficiently calculate the distance matrix for the pair- or cross-correlation of SMLM data across the entire field of view (Figure 1). Based on the cross-correlation, a distance cutoff can be defined to separate localizations that cluster or colocalize with a second protein of interest. These separated molecule lists can then be separately visualized and further analyzed with any existing secondary analysis algorithm to e.g. determine the number of molecules in and the size of a cluster, their diffusive state in live-cell data, or their degree of cross-correlation (Owen et al., 2010; Sengupta et al., 2011; Veatch et al., 2012; Puchner et al., 2013; Stone and Veatch, 2015; Pageon et al., 2016a, 2016b; Hummer et al., 2016; Lagache et al., 2018; Banerjee et al., 2020; Heydarian et al., 2021; Marenda et al., 2021). We first demonstrate the performance and application of this method with simulated data sets to allow a comparison of the results to a known ground truth. We also show the application to two-color SMLM data of ULK1 and Atg13, two proteins that have been recently shown to be involved in the initiation of autophagy when co-clustered. Since our described method can be paired with any existing downstream SMLM data analysis algorithm, it presents a useful and modular way to improve SMLM analysis results e.g. by suppressing randomly localized noise localizations and by providing a refined comparison between clustered and non-clustered localization.

## Materials and Methods

### Workflow

In our code, available at https://github.com/PuchnerLab/cross-correlation-filtering, the point of entry is the MATLAB function “cc_graphic_pipeline”, which accepts as arguments each list of coordinates (as x-y columns), the maximum distance used for the pair- and cross-correlation calculations, the area of the field of view of the localization data (for correct normalization of the cross-correlation), and the units in which the data is provided. This function outputs the pair-correlation of each dataset and the cross-correlation between the two datasets. From the generated graphs, the user can determine appropriate cutoff distances for cross-correlation filtering and for the optional clustering. As an aid, the pair-correlations indicate the distance to the 99% drop in correlation, and the cross-correlation indicates the distance to both the 50 and 99% drop in correlation.

The second step is the function “cc_separation_pipeline”, which accepts as arguments each list of coordinates, a vector of cutoff distances for clustering for each dataset [(0, 0) if no clustering is to be performed], the cutoff distance for the cross-correlation filtering, and a vector of minimum stoichiometries considered for colocalization for each dataset [(1, 1) for no minimum]. The primary output is a cell with each element containing a logical vector of colocalized localizations of each dataset, which can be used to select the colocalized and non-colocalized localizations from the original datasets or from the indices provided by another cluster assignment algorithm, such as DBSCAN (Ester et al., 1996). Additionally, “cc_separation_pipeline” outputs a cell of the colocalized coordinates and a cell of non-colocalized coordinates.

In this second step, localizations from the two coordinate lists that lie within the cutoff distance are assigned as colocalized. If the optional clustering is performed prior to colocalization, then two clusters are assigned as colocalized if any of their constituent localizations are within the cutoff distance and if the number of localizations in the cluster is at least the specified minimum number of localizations for each cluster.

A schematic of the organization of the code is shown in Supplementary Figure S1.

### Simulated Data

**FIGURE 2:**
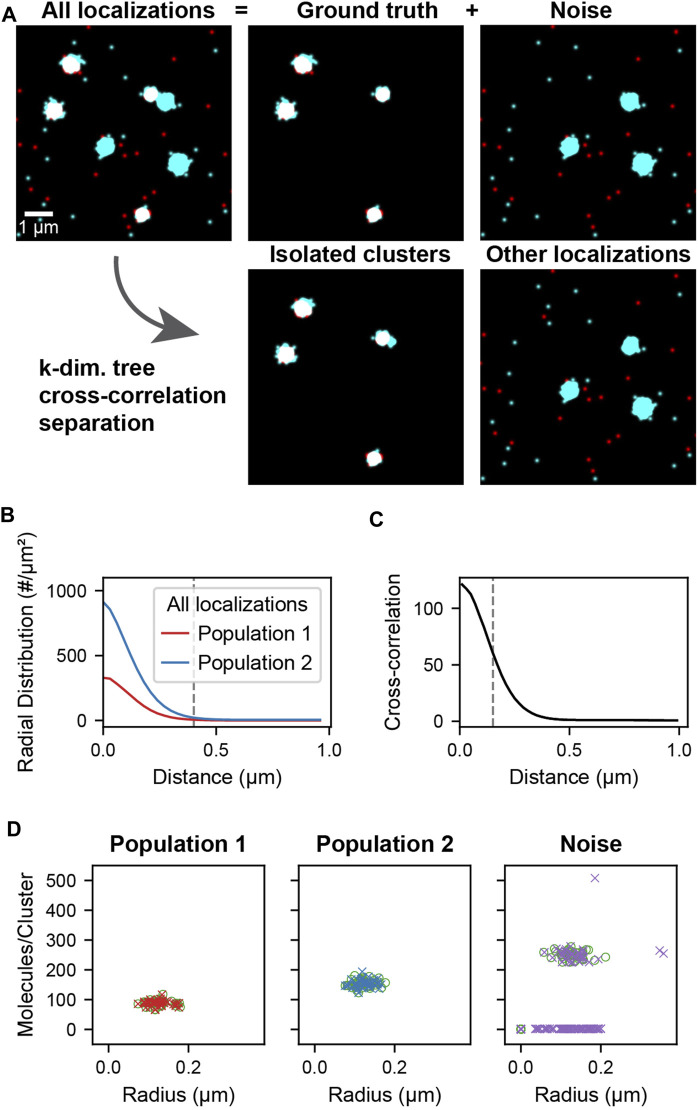
Cluster separation on simulated data. A data set was simulated consisting of two types of localizations as well as added noise. Population 1 (red) and 2 (cyan) consists of 40 clusters of size 0.1 ± 0.02 µm and stoichiometries of Poisson (90) and Poisson (150) respectively and represents the ground truth. Noise localizations include non-colocalized clusters of size 0.1 ± 0.02 µm and stoichiometry Poisson (250) (cyan) as well as randomly distributed localizations (red) to measure the performance of the cluster separation. **(A)** Super-resolution reconstruction of all localizations, the ground truth clusters of population 1 and 2, and the noise localizations (upper). After applying the distance-based cutoff, the two molecule lists can be separated into the colocalized clusters and all other remaining noise localizations (lower). **(B)** Pair correlation functions of each of the two ground truth populations showing the relevant length scale of cluster sizes used to identify clusters. **(C)** Cross-correlation functions of all localizations between the two populations for all localizations. The dashed line indicates the maximum separation two localizations can have to be classified as colocalized. If two localizations that belong to clusters as identified in B are closer than this maximum separation, all localizations from the entire clusters are classified as colocalized. **(D)** Stoichiometry and size of ground truth (circles) and recovered clusters (crosses) for each of the populations.

The simulated localization data used in cross-correlation filtering (Figure 2A, All localizations) is composed of two parts: the ground truth clusters (Figure 2A, Ground truth) and noise clusters and localizations (Figure 2A, Noise). The clusters were generated by randomly distributing cluster centers throughout the field of view. Localizations were placed by generating coordinates from a normal distribution around each cluster center. Localizations were generated in this way for both populations of clusters using the same cluster centers so that colocalized clusters have complete overlap (Figure 2A, Ground truth). Randomly distributed localizations were mixed into each population to simulate localizations that are not clustered or colocalized. Additional clusters of higher stoichiometry were mixed into the second population to simulate non-colocalized clusters that should be separated by the analysis (Figure 2A, Noise). A schematic of the simulated data construction is show in Supplementary Figure S2. The recovery of the underlying colocalized ground truth and noise rejection was quantified by computing the F-score as a function of the colocalization cutoff distance (Supplementary Figure S3A). The recovery of the correct radii and stoichiometries was quantified in Supplementary Figure S3B and Supplementary Figure S3C, respectively.

#### Cross-Correlation and Cluster Separation Analysis

In each population of simulated data, localizations appearing within a distance of 0.4 µm from each other were first assigned to clusters. Next, a cross-correlation analysis was performed by using a k-dimensional tree for fast querying and to limit the memory consumption of the distance tabulation. A range search was then performed to compute the distances between each localization of one population and those of the other population up to a specified maximum separation distance, which was chosen to be 1 µm. The indices and distances from the range search are used to determine which clusters from one population are colocalized with clusters from the other population. A maximum separation of 150 nm between the constituent molecules of two clusters was used for determining their colocalization and at the same time a requirement of a minimum of two localizations per cluster was imposed to filter out individual non-colocalized localizations. The resulting lists of colocalized clusters (and complementary list of excluded clusters and localizations) were then further analyzed to determine their stoichiometry and size compared to the ground truth clusters. The performance of the colocalization analysis was also quantified on lines and rings (Supplementary Figure S6).

### Benchmarks

To test the memory and time efficiency of the k-dimensional tree-based cross-correlation compared to full distance matrix approach, we simulated two populations of completely colocalized clusters of stoichiometries of 200 and 300 localizations normally distributed about the centroid with standard deviation 100 nm and 10,000 individual noise localizations in each population within a 40.96 µm field of view. We varied the total number of clusters within the field of view to increase the memory requirements of the cross-correlation.

#### Memory Efficiency

**FIGURE 3:**
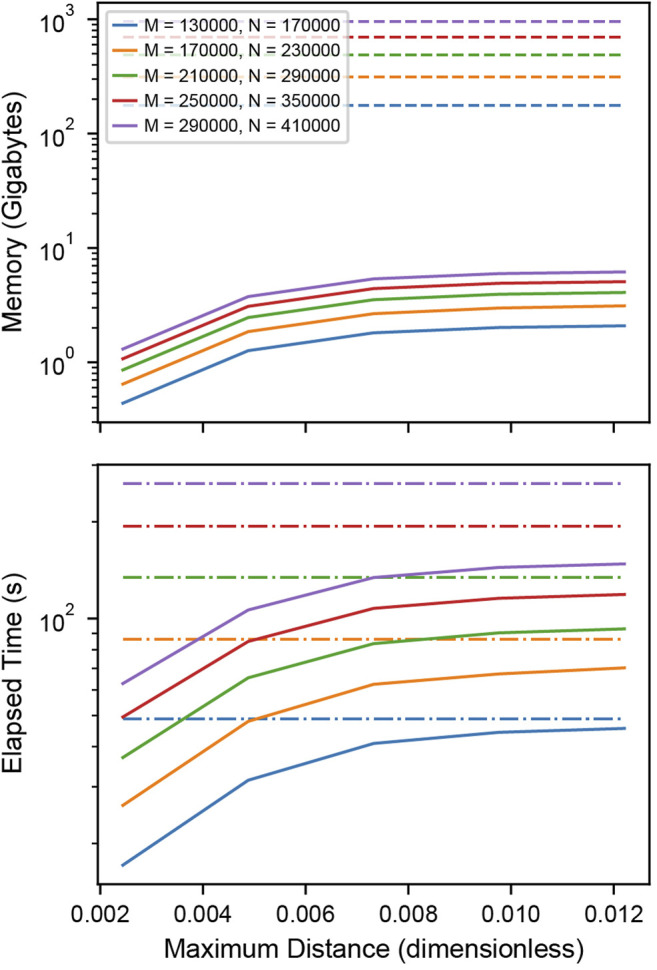
Performance of distance tabulation using a k-d tree and range search vs. a full distance matrix. **(A)** Simulated data sets as in Figure 2 with varying numbers of localizations were analyzed to measure the memory needs for calculating the full distance matrix (dashed) and k-dimensional tree (solid) with distance cutoff. For matrices that could not explicitly be allocated into memory, a linear extrapolation was applied to estimate the allocation time (dot-dashed). The total number of clusters is increased with an increasing number of localizations to increase the memory requirements of the distance tabulation. The distance is expressed as a fraction of the field of view. For relevant length scales, the tree uses significantly less memory than the matrix. **(B)** Elapsed time for calculating the full distance matrix (dashed) vs. the k-dimensional tree (solid). When a realistic number of localizations is included in the distance tabulation, the distance tabulation for the tree takes less time than the full matrix.

Memory requirements in Figure 3 A were determined by calculating the total number of bytes of memory needed for storing the distances between the localizations. For the matrix-based analysis, this is MNB , which corresponds to the number of elements in the distance matrix, where M and N are the number of localizations in each population and B is the number of bytes corresponding to the floating-point precision used. For the k-dimensional tree-based analysis, the total amount of memory is bounded from above by MNB , gradually approaching it as the range is increased. For structure sizes much smaller than the field of view, the k-dimensional tree distance tabulation outperforms the distance matrix when including only the relevant distances and excluding potentially less relevant long-range distances. In the cases where the matrix memory requirements did not exceed the system memory (as in Supplementary Figure S5A), the memory consumption was measured directly.

#### Time Efficiency

Elapsed times in Figure 3 B and Supplementary Figure S5B were determined by measuring the time needed to construct either the full distance matrix or the range search on the k-dimensional tree for various distances. For matrices with a memory allocation that would exceed the available system memory, the allocation time was extrapolated from the allocation rate determined by fitting the matrix allocation vs memory for moderate data (Supplementary Figure S5D).

### Mammalian Cell Analysis

**FIGURE 4:**
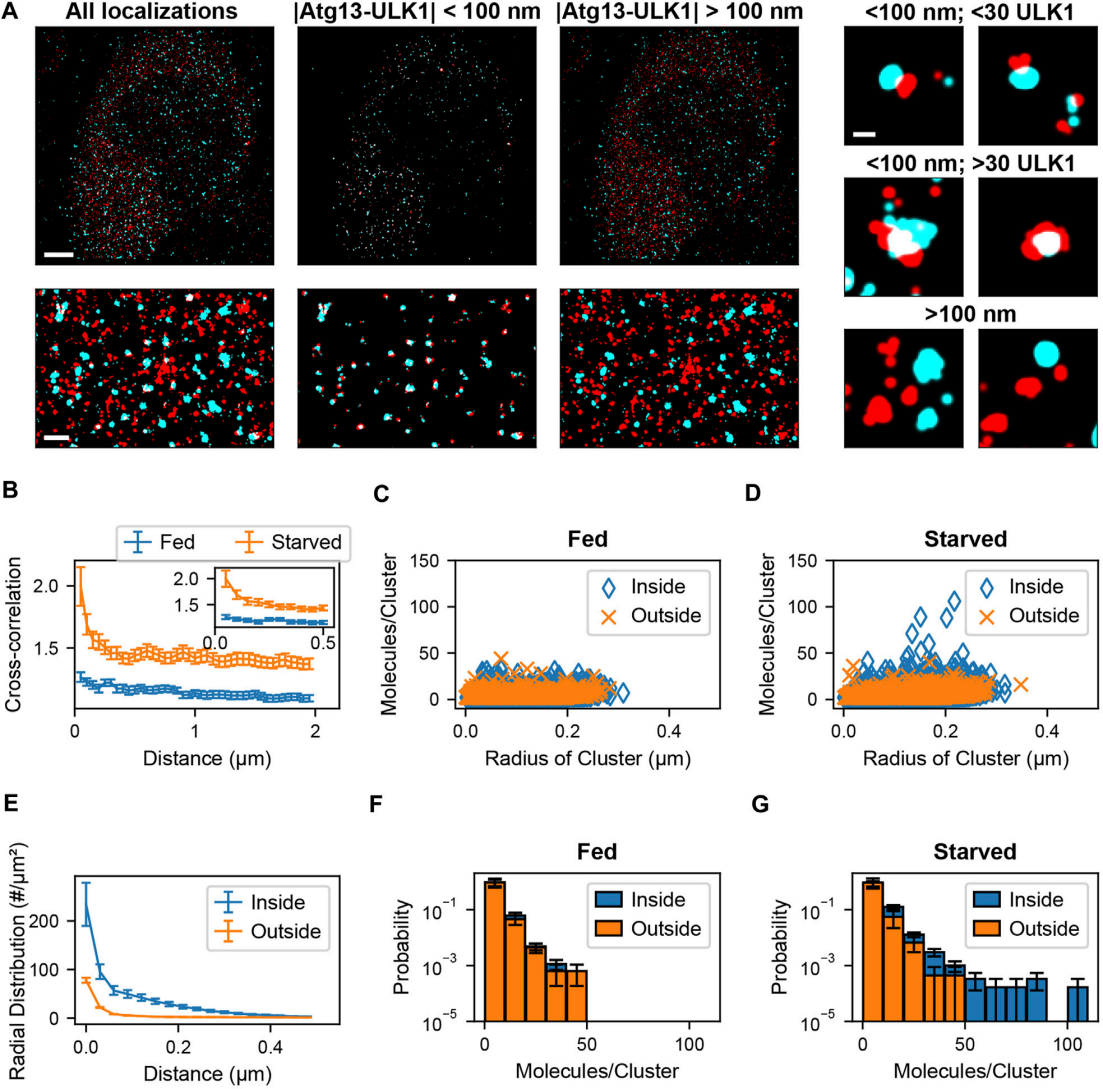
Colocalization analysis of ULK1 and ATG13 via cross correlation. **(A)** Left shows a two-color overlay of PALM images of mEos2-ULK1 (red) and HaloTag-ATG13 bound to JF646 (cyan) with a corresponding magnified image (bottom left) taken from a representative starved cell (Scale bar top left: 5 µm; bottom left: 1 µm). Middle represents the ATG13 localizations that are within 100 nm of ULK1 molecules and considered colocalized with ULK1 clusters. The corresponding magnified image (bottom middle) highlights the increased visibility of colocalized ULK1 clusters after filtering out non colocalized molecules using the cross-correlation analysis framework. Right represents ULK1 and ATG13 localizations that are further than 100 nm away from each other and are not considered colocalized. Examples of individual clusters that are and are not colocalized with ATG13 localizations are also displayed (scale bar: 150 nm). **(B)** Cross-correlation plot between ULK1 molecules and ATG13 localizations in fed (blue) and starved (red) cells. The cross correlation plot was calculated by using k dimensional trees to obtain ATG13 localizations within 2 µm of ULK1 molecules across the entire field of view. The inset graph represents the onset of leveling of the cross correlation plot around 100 nm. The error bar corresponds to SEM from five fed and five starved cells. **(C,D)** show the quantification of the number of molecules and radii of ULK1 clusters colocalized with ATG13 (inside, blue) and not colocalized with ATG13 (outside, orange) in fed **(C)** and starved **(D)** cells. ULK1 localizations that are and are not colocalized with ATG13 exhibited similar distributions in fed cells while ULK1 localizations that colocalized with ATG13 in starved cells formed structures that contained more molecules and were interpreted to be forming autophagosomes. **(E)** The radial distribution function further quantifies the local density difference between ULK1 colocalized (inside, blue) vs. non ATG13 colocalized (outside, red) ULK1 molecules in starved cells. The error bar represents SEM from five starved cells. Figure **(F)** and **(G)** display a normalized histogram of the number of ULK1 molecules in a cluster colocalized with ATG13 (inside, blue) and not colocalized with ATG13 (outside, orange) in fed **(F)** and starved **(G)** cells. The error bar corresponds to SEM from five fed and five starved cells. While there was no noticeable difference in ATG13 colocalized and non ATG13 colocalized distributions in fed cells, there was a significant difference in both distributions in starved cells where ATG13 colocalized clusters contained the highest number of ULK1 molecules.

Sample preparation, imaging methods, and blink correction analysis are described in Banerjee et al., 2020 (Banerjee et al., 2020). After identification of blink corrected ULK1 molecules, molecules were assigned to clusters. The radial distribution (or pair correlation) among all blink corrected ULK1 molecules with respect to each other was calculated. The leveling of the pair correlation plot approached zero at around 400 nm (Banerjee et al., 2020). Nearby ULK1 molecules whose distances lie within the cutoff distance of 400 nm were assigned to the same ULK1 cluster. After this, a spatial cross-correlation between the blink corrected ULK1 molecules and ATG13 localizations was calculated to determine the pairwise distance distribution between the two protein populations. To overcome memory limitations associated with existing methods, the ATG13 dataset was converted into a k-dimensional tree structure as described in the results section. Then, a nearest neighbor search was used to obtain pairwise distances between ULK1 and ATG13 molecules up to a specified distance cutoff of 2 µm. A cross correlation function was then calculated between the obtained ATG13 and ULK1 pairwise distances using previously described methods (Veatch et al., 2012; Banerjee et al., 2020). Since the cross-correlation curve remained constant at distances larger than 100 nm, which indicates no clustering beyond this distance, this number was used as the colocalization distance cutoff (Figure 4B). ATG13 localizations within the 100 nm distance cutoff of ULK1 molecules were therefore considered colocalized with ULK1 molecules. ULK1 and ATG13 molecules were segregated into colocalized and non-colocalized groups. Cell and matrix array computations were parallelized to increase computational speed. Next, ULK1 clusters that contained at least one molecule colocalized with an ATG13 localization was identified as a colocalized cluster. These clusters were separated from non-ATG13 colocalized clusters and further analyzed. Cluster properties such as radius and the number of molecules for both ULK1 clusters were then obtained. Radial distribution functions of various sub ULK1 cluster populations were calculated (ATG13 colocalized vs non-ATG13 colocalized molecules) by normalizing the separated pairwise distances by the bin area and the number of molecules in those individual datasets. Cluster properties and pairwise distance distributions from the full cell and isolated sub-populations within the cell were pooled together to compare how these metric change between fed and starved cells. The analysis codes were written in MATLAB 2018b and run on a Dell PowerEdge T440 server with 94GB RAM, an Intel Xeon 2.68 GHz CPU, and 14.5 TB of disk space.

## Results

### Cluster Separation to Remove Noise From Simulated Data

The cross-correlation and cluster separation analysis can provide information about the stoichiometry and size of colocalized clusters while filtering out localizations that do not belong to clusters or are not colocalized. To demonstrate the application and to measure the performance of this analysis, we first simulated localization data, allowing us to compare the processed results to the ground truth. Figure 2 shows the simulated cluster data for two populations of localizations. The ground truth consists of randomly distributed clusters of population 1 with a mean number of 90 localizations that colocalize with clusters of population 2 that have a mean number of 150 localizations. Superimposed to the ground truth are randomly distributed localization of population 1 as well as randomly distributed localizations and clusters of population 2 with a mean of 250 localizations. In this way, non-colocalized localizations and clusters are introduced as noise, which is meant to be filtered by our analysis pipeline. First, localizations in each population are assigned to a cluster if they appear within 0.4 µm of each other, which corresponds to the peak-width of the pair-correlation and reflects the average cluster size (Figure 2B). Next, colocalized clusters from both populations were identified based on the cross-correlation if two localizations were separated by less than 150 nm (Figure 2C, Supplementary Note S1, Supplementary Figure S4). As can be seen in Figure 2A, the isolated clusters and remaining localizations reflect to a large degree the original ground truth and noise localizations. By determining the number of localizations in each of the colocalized cluster, the original distribution that was used for the simulation is recovered to above 98% for population 1 and above 93% for population 2 (Figure 2D, Supplementary Figure S4). Likewise, further structural analysis of the isolated clusters such as the determination of cluster radii recovers the same results from the ground truth. Overall, 100% of the 40 ground truth clusters in each population were recovered and most deviations arose from noise localizations and clusters that are coincidentally in proximity to the ground truth. These results demonstrate that our cross-correlation based approach is effective in separating noise localizations and clusters from colocalized clusters for further downstream analysis.

### Benchmarks

For large enough SMLM datasets, constructing a full distance matrix for a cross-correlation analysis can approach or even exceed the available system memory. This is because distances between all localizations across the field of view are tabulated, including distances that are well beyond relevant length scales of clusters or structures under investigation. Using the k-dimensional tree-based approach to compute the cross-correlation can significantly reduce the memory requirements by orders of magnitude when an appropriate cutoff distance is selected for tabulating the distances. Figure 3A shows how the memory requirements of the k-dimensional tree-based method increase with the cutoff distance and remain orders of magnitude below the full matrix for relevant length scales. As the cutoff distance increases up to the full field of view, the memory consumption approaches that of the full distance matrix. Figure 3B shows that the computation time is worse for the k-dimensional tree-based method when applied to smaller datasets but eventually shows improved runtime performance as the size of the dataset increases and reaches more realistic numbers in the range of thousands of localizations. When extrapolating the memory consumption to 100 GB, which is at the order of magnitude where the calculation becomes infeasible, a number of 112,000 localization is obtained for the full distance matrix and 44, 200, 000 for k-dimensional trees at a distance cutoff of 0.5 µm (Supplementary Note S2, Supplementary Figure S5C). This result demonstrates that typical SMLM data sets cannot be analyzed with a full cross-correlation and that significantly larger data sets can be analyzed using k-dimensional trees.

### Isolating Co-clustered ULK1 and Atg13 Structures That Form Phagophores

In the following we demonstrate the isolation of co-clustered structures in real two-color PALM data that recently led to the identification of signaling clusters involved in autophagy (Banerjee et al., 2020). Autophagy is a subcellular process in eukaryotic cells in which macromolecules and organelles are engulfed by a double membrane and then degraded by fusion with lysosomes (Mercer et al., 2009; Jung et al., 2010; Roach, 2011; Park et al., 2018). Autophagy can be induced by amino acid starvation and the resulting inhibition of mechanistic target of rapamycin complex 1 (mTORC1) (Kamada et al., 2000; Chang and Neufeld, 2009, 1), which in turn leads to the formation of autophagy initiation cluster composed of activated UNC51-like kinase 1 (ULK1), Atg13, FIP200 (FAK family kinase interacting protein 200 kDa), and Atg101 (Hosokawa et al., 2009). In a recent study we employed CRISPR/Cas9 based genome editing to endogenously tag ULK1 with the photoswitchable fluorescent protein mEos2 in HeLa cells and to perform a quantitative PALM colocalization analysis with Atg13, a critical interaction partner of ULK1 in activation cluster (Banerjee et al., 2020). Our results showed that amino acid starvation induced the formation of a small fraction of arc shaped and spherical structures containing more than 30 ULK1 molecules that all colocalized with Atg13 in proximity to the Endoplasmic Reticulum. Therefore, a threshold number of ULK1 is required to initiate the formation of autophagosomes. Here, we demonstrate the application of our cross-correlation approach to a modified data sets similar to the ones shown in (Banerjee et al., 2020).

Two-color PALM data was recorded using endogenously tagged ULK1-mEos2 and transiently transfected Atg13-HaloTag in conjunction with the PALM compatible JF646 dye as described in (Banerjee et al., 2020). As can be seen in Figure 4 A in both fed and starved cells ULK1 and Atg13 formed puncta that did and did not colocalize. However, in starved cells a few larger structures with a higher number of ULK1 molecules are visible. Due to the large number of localizations in the 5 data sets (9,589 ± 904 ULK1 molecules, 1,270,045 ± 420,310 Atg13 localizations), a traditional cross-correlation across the entire fields of view is not feasible with commonly used computers or servers (our server (2.68 GHz CPU, 94 GB RAM)—typical computer (2.7 GHz CPU and 8–20 GB RAM) since it would require up to 95 GB of RAM memory. We therefore employed k-dimensional trees to efficiently calculate the distance matrix for the cross-correlation across the entire field of view (Figure 4B). The cross-correlation between ULK1 and Atg13 was significantly larger in starved cells and exhibited a pronounced peak up to distances of ∼100 nm, indicating the formation of more densely colocalized structures. Using the cross-correlation matrix, it is now possible to separate the molecule list of ULK1 and Atg13 localizations that are closer than 100 nm and considered to be colocalized. All remaining localizations that are separated by more than 100 nm are accumulated in a separate molecule list (Figure 4C). Importantly, these separated molecule lists can now be further processed with any secondary SMLM data analysis approach. For instance, when plotting the number of ULK1 molecules in clusters against the radius of structures, it becomes apparent that under starvation a unique but rare population of structures with a large number of ULK1 molecules emerges (Figure 4D). Importantly, this population of structures is not present in fed cells and always colocalizes with Atg13. Based on this result and further evidence provided in (Banerjee et al., 2020), these structures are identified to be involved in autophagy and the formation of autophagosomes.

Another commonly used secondary data analysis approach is the use of pair-correlation or radial distribution functions to determine the average density of pairs of localization with respect to their distance (Puchner et al., 2013; van Lengerich et al., 2017; Banerjee et al., 2020). We therefore calculated the radial distribution function of ULK1 localizations that did and did not colocalize with Atg13. ULK1 localizations that did not colocalize with Atg13 exhibited the lowest density and only a small peak at short distances up to ∼74 molecules/µm^2^ in starved and fed cells, indicating a small degree of basal clustering in the absence of Atg13 (Figure 4E). Since no significant difference is observed between fed and starved cell, these clusters are interpreted to be passive and not involved in autophagy initiation. ULK1 structures that did colocalize with Atg13 exhibited a significantly larger ULK1 density up to ∼234 molecules/µm^2^ in starved cells (Figure 4E). Since this density is also significantly larger than in fed cells, these clusters are interpreted to be dense initiation clusters that form in response to starvation and aid in the formation of phagophores (Banerjee et al., 2020) for details. Histograms of the ULK1 stoichiometry show that ULK1 clusters colocalized and not colocalized with Atg13 have identical stoichiometries in the fed case (Figure 4F) but colocalized ULK1 clusters have a higher stoichiometry in the starved case (Figure 4G). These results demonstrate that our presented SMLM analysis approach is powerful to isolate protein clusters and nanoscopic structures that are rare but of biological significance. Furthermore, any secondary SMLM data analysis such as quantification of molecule numbers, densities or sizes of structures can be applied to the isolated molecule lists to gain further insight into the nanoscopic characteristic and differences between different populations.

### Comparison to Existing Colocalization Methods

To demonstrate the advantages k-d tree-based cross-correlation, we performed a comparison to existing methods that employ radial threshold-based colocalization, density-based cluster detection, and tessellation-based cluster detection/colocalization. The MATLAB based Clus-DoC program (Pageon et al., 2016a) combines a radial threshold-based method to characterize colocalization with a density-based cluster detection to identify colocalized molecules. This method first utilizes Ripley’s K analysis to calculate the radial distance distribution between the colocalized molecule populations and then assigns each localization a normalized score based on its proximity to surrounding localizations of the opposite molecule population. The algorithm sets a score cutoff that is akin to a radial distance threshold to determine whether individual molecules are colocalized. Then, Clus-DoC uses the density-based clustering algorithm DBSCAN to segment localizations into individual clusters. Both techniques are commonly used in a variety of other clustering and colocalization algorithms (Ester et al., 1996; Owen et al., 2010; Malkusch et al., 2012; Lagache et al., 2018). The algorithm then combines both approaches to separate colocalized clusters with a minimum number of localizations from non-colocalized clusters. Finally, the algorithm calculates size and density metrics of colocalized clusters. This algorithm is most similar to our approach as it employs a radial distance distribution analysis to characterize the degree of colocalization between two protein populations and defines a threshold based on the radial distance to isolate colocalized molecules/cluster for downstream analysis. When validated against simulated datasets shown in Figure 2 and described in the methods section, we find that the accuracy metrics are similar to ours since cross-correlation analysis is similar to Ripley’s K analysis (Table 1) and since both methods rely in part on a distance threshold derived from the distance distribution. The main advantage our method compared to the Clus-DoC approach is the efficient analysis of the entire field of view of large datasets. Though Clus-DoC employs k-d trees to calculate the Ripley’s K distribution, it requires the calculation of the full distance matrix to segment molecules into clusters and to isolate colocalized clusters. Therefore, Clus-DoC cannot analyze the full field of view for large 340 gigabyte-1.1 terabyte datasets due to large memory requirement (Table 1). In contrast, our method is able to efficiently analyze the full field of view of 340 gigabyte-1.1 terabyte sized datasets while needing a fraction of the available memory (Table 1). The memory required for our largest 1.1 TB simulated dataset is 4.7 GB which is similar to the memory available on laptops. Furthermore, using k-d trees for pair correlation analysis allows us to efficiently calculate the distance distribution across the full field of view to make an accurate assessment of the cutoff distances needed to segment molecules into clusters. Our k-d tree-based colocalization analysis also has an improved run time when compared to Clus-DoC (Table 1).

**TABLE 1:** Performance Comparison of existing methods. This table compares our proposed kd-tree approach to three existing approaches, Clus-DoC, Coloc-Tesseler, and full matrix approach. Clus-DoC utilizes Ripley’s K analysis and density based clustering using DBSCAN to segment localizations into clusters and separate colocalized from non-colocalized clusters. Coloc-Tesseler uses Voronoi tessellations to assess whether molecules are co localized and uses the tessellation diagram to draw boundaries around colocalized clusters. Datasets were simulated in a similar manner to those shown in Figure 2 and described in the methods section. Time was measured as the entire time required to run program after data files were load. An F-score (described in the methods section) was used to compare colocalization accuracy among all datasets. Since localization lists cannot be outputted from Coloc-Tesseler, the colocalization accuracy of this method cannot be calculated. Furthermore, due to large memory requirement, Clus-DoC and the full matrix method cannot analyze datasets above 94 GB. Memory use by Coloc-Tesseler was estimated from task manager since it is a GUI based executable program with no available source code but all simulated molecule lists could be analyzed in an efficient time window (seconds) while only requiring between 0.2 and 1.5 GB of memory.

Number of localizations in population 1	Number of localizations in population 2	Full matrix memory (GB)	Clus-DoC memory (GB)	Coloc-Tessler memory (GB)	k-d tree memory (GB)
20,000	25,000	4	4	0.21	0.15
30,000	40,000	9.6	9.6	0.22	0.31
50,000	70,000	28	28	0.43	0.61
70,000	100,000	56	56	0.58	0.91
170,000	250,000	340	Not possible to compute	0.78	2.45
250,000	370,000	740	Not possible to compute	0.85	3.74
310,000	460,000	1,140	Not possible to compute	1.42	4.70
**Number of localizations in population 1**	**Number of localizations in population 2**	**Full matrix time (s)**	**Clus-DoC time (s)**	**Coloc-Tessler time (s)**	**k-d tree time (s)**
20,000	25,000	30.5	48.26	1.35	7.11
30,000	40,000	63.7	103.42	1.53	8.47
50,000	70,000	138.2	340.26	2.57	15.88
70,000	100,000	289.2	739.30	3.54	23.36
170,000	250,000	Not possible to compute	Not possible to compute	9.72	70.45
250,000	370,000	Not possible to compute	Not possible to compute	25.3	95.39
310,000	460,000	Not possible to compute	Not possible to compute	42.54	120.38
**Number of localizations in population 1**	**Number of localizations in population 2**	**Full matrix accuracy**	**Clus-DoC accuracy**	**Coloc-Tessler accuracy**	**K-d tree accuracy**
20,000	25,000	0.99	0.98	Not possible to compute	0.98
30,000	40,000	0.99	0.99	Not possible to compute	0.99
50,000	70,000	0.99	0.99	Not possible to compute	0.99
70,000	100,000	0.99	0.99	Not possible to compute	0.99
170,000	250,000	Not possible to compute	Not possible to compute	Not possible to compute	0.99
250,000	370,000	Not possible to compute	Not possible to compute	Not possible to compute	0.99
310,000	460,000	Not possible to compute	Not possible to compute	Not possible to compute	0.99

We also compared our method to colocalization analysis approaches based on Voronoi tessellation. These approaches have gained popularity since the detection of colocalized localizations does not require as a radial distance threshold, Ripley’s K score, or density threshold (Levet et al., 2015, 2019; Andronov et al., 2016). Instead, these algorithms use Voronoi tessellations to determine cluster boundaries by using the relative similarities in the areas of polygons and densities of localizations. These techniques also allow for the direct calculation of Spearman’s rank coefficients and Mander’s coefficients to quantify the degree of clustering in the same molecule population and the degree of colocalization between multiple molecule populations. Coloc-Tesseler (Levet et al., 2019) is a graphical user interface (GUI) based C++ program that uses Voronoi tessellations to assess colocalization. Molecule lists from both molecule populations are directly inputted into the program through the GUI. The program then outputs colocalized molecules of one population, colocalized molecules of the other population, and non-colocalized molecules of both populations. The Voronoi diagram visually highlights the density difference between the colocalized and non colocalized population. Mander’s and Spearman’s rank coefficients can be calculated for a defined region of interest with the plot to quantify the degree of colocalization in that area. The user is also able to further refine co localization performance by altering relative density cutoffs used by the program to define cluster boundaries. This program can analyze large simulated datasets quickly while having a similar memory requirement as our k-d tree-based program (Table 1). The main drawback is that colocalized molecule lists or colocalized cluster lists cannot be outputted by the program which makes downstream analysis impossible. In addition, an accuracy analysis via an F-score, which requires true positives, false positives, false negatives, and true negatives cannot be calculated. The only other program outputs besides the color coded Voronoi plot are a quantification of colocalization via Mander’s and Spearman rank coefficients. However, these metrics do not contain information about the distance dependent degree of colocalizations that the cross-correlation methods directly quantify. This distance dependent degree of-colocalization is particularly useful when comparing datasets across different states such as comparing the degree of colocalization between fed and starved cells at various distances (Figure 4).

In summary, while Voronoi tessellation efficiently detects clustering and colocalization of SMLM data, it does not contain the distance dependent density information of cross-correlation methods, which is useful for comparing data sets and for separating colocalized molecule lists for further downstream analysis. Our implementation of k-d trees for calculating the auto- or cross-correlation significantly lowers the computational time and memory needs, which allows for the analysis of large SMLM data sets that cannot be analyzed with existing cross-correlation methods. The modular code can be interfaced from existing SMLM data analysis packages for up- and downstream analysis and therefore enables the detection of otherwise hidden features such as the critical number of ULK1 molecules in rare clusters that initiate autophagy.

## Data Availability

The data analyzed in this study is subject to the following licenses/restrictions: Simulated data from Figure 2 is available in the code repository located at https://github.com/PuchnerLab/cross-correlation-filtering Due to the large size of the SMLM data sets, they will not be uploaded but made available upon request. Requests to access these datasets should be directed to EMP, epuchner@umn.edu.
