# Supplementary material for: Efficient Cross-Correlation Filtering of One- and Two-Color Single Molecule Localization Microscopy Data
Source: Front Bioinform. 2021 Nov 4;1:739769. doi: 10.3389/fbinf.2021.739769 (PMC9581065; doi:10.3389/fbinf.2021.739769)
Supplement: Supplementary file 1 [file DataSheet1.docx]

Supplementary Material

Efficient cross-correlation filtering of one- and two-color single molecule localization microscopy data

Angel Mancebo^1🕆^, Dushyant Mehra^1,3🕆^, Chiranjib Banerjee^1^, Do-Hyung Kim^2^, Elias M. Puchner^1*^

^1^School of Physics and Astronomy, University of Minnesota, Twin Cities, Minneapolis, MN, United States

^2^Department of Biochemistry, Molecular Biology, and Biophysics, University of Minnesota, Twin Cities, Minneapolis, MN, United States

^3^Department of Physiology and Biomedical Engineering, Mayo Clinic, Rochester, MN, United States

^🕆^ **Equal Contribution**

*** Correspondence:**Corresponding Author
epuchner@umn.edu

**Supplementary Note 1:**

To demonstrate how the cross-correlation changes with the degree of colocalization between two clusters, we consider the case of two identical Gaussian distributions with a varying distance between them. For a two-dimensional Gaussian distribution

|  | $f\left( x, y;\sigma\right)=\frac{1}{\pi\sigma^{2}}\exp\left( -\frac{\left( x^{2}+y^{2} \right)}{2\sigma^{2}} \right)$ | Eq 1 |
| --- | --- | --- |

the cross-correlation between two clusters with a separation $d$ can be computed by

|  | $xcorr\left( u,v \right)=f\left( x,y;\sigma_{1} \right)\star f\left( x-d,y;\sigma_{2} \right)$ | Eq 2 |
| --- | --- | --- |
|  | $=\int_{-\infty}^{\infty} dx\int_{-\infty}^{\infty} dyf\left( x,y;\sigma_{1} \right)f\left( x-d+u,y+v;\sigma_{2} \right)$ | Eq 3 |
|  | $=\frac{2}{\pi\left( \sigma_{1}^{2}+\sigma_{2}^{2} \right)}\exp\left( -\frac{\left( u^{2}+v^{2}-2ud+d^{2} \right)}{2\left( \sigma_{1}^{2}+\sigma_{2}^{2} \right)} \right)$ | Eq 4 |

To project this to one dimension and produce a cross-correlation plot, we rewrite this with $u=r cos(\theta)$ and $v=r sin(\theta)$ and integrate through all angles. The cross-correlation becomes

|  | $xcorr\left( r \right)=\frac{1}{2\pi}\int_{0}^{2\pi} d\theta\frac{2}{\pi\left( \sigma_{1}^{2}+\sigma_{2}^{2} \right)}\exp\left( -\frac{\left( r^{2}+d^{2}-2rdcos \left( \theta\right) \right)}{2\left( \sigma_{1}^{2}+\sigma_{2}^{2} \right)} \right)$ | Eq 5 |
| --- | --- | --- |
|  | $=\frac{2I_{0}\left( \frac{rd}{\sigma_{1}^{2}+\sigma_{2}^{2}} \right)}{\pi\left( \sigma_{1}^{2}+\sigma_{2}^{2} \right)}\exp\left( -\frac{\left( r^{2}+d^{2} \right)}{2\left( \sigma_{1}^{2}+\sigma_{2}^{2} \right)} \right)$ | Eq 6 |

where $I_{0}(\cdot)$ is the modified Bessel function of the first kind of order zero.

Figure S4 shows the scaled cross-correlation of two clusters of $\sigma_{1}=\sigma_{2}=$ 0.1 µm and separation $d$ of up to 1 µm.

**Supplementary Note 2:**

To extrapolate the memory consumption of the distance tabulation and determine the number of localizations that would make the calculation infeasible, we consider the memory required for a distance tabulation by an explicit distance matrix, which is

|  | $matrix=MNB=(mc+p)(nc+p)B$ | Eq 7 |
| --- | --- | --- |

where $c$ is the number of clusters, all taken to be colocalized, $n,m$ are the number of localizations in each cluster within each population, and $p$ is the number of individual noise localizations. For the range search, the distance is truncated such that only distances between colocalized clusters are tabulated and not distances between distal clusters. Therefore, at the cutoff distance, the total memory consumption becomes

|  | $range=\left( mnc+\left( m+n \right)cq+q^{2} \right)B$ | Eq 8 |
| --- | --- | --- |

Where $q=p\frac{\pi d^{2}}{A}$, the number of noise localizations that are by chance included in the distance tabulation, with $d$ the diameter of the search radius and $A$ the area of the field of view. Figure S5C shows the extrapolation compared to measured memory allocation data for simulated clusters with noise.

**
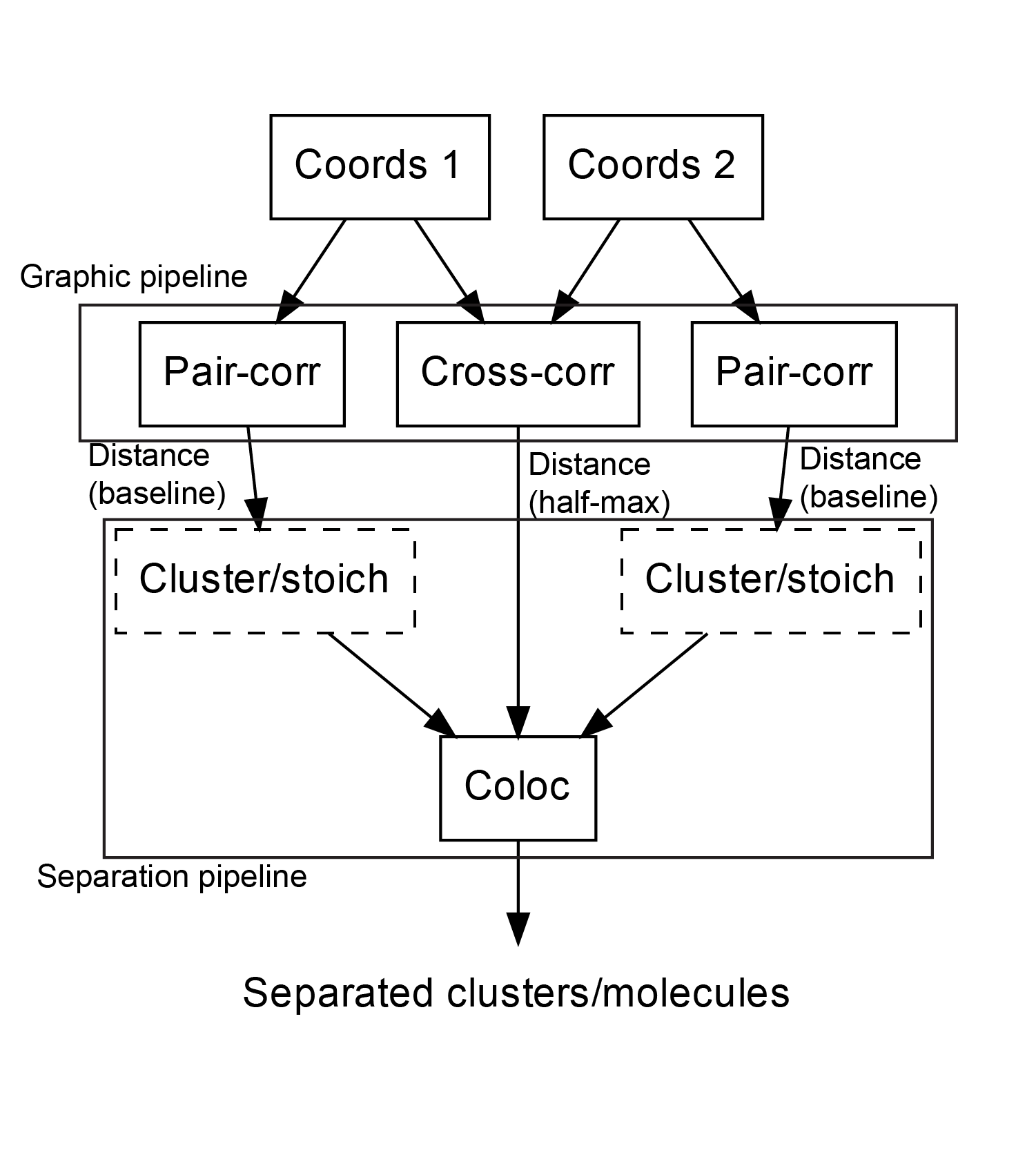
**

**Supplementary Figure 1: Schematics of the analysis code.** The two sets of coordinates are passed into the k-dimensional tree based cross-correlation function to determine an appropriate distance to use for their colocalization, based on the half-maximum of the correlation function. If each data sets is analyzed for clustering individually to establish stoichiometry restrictions on the colocalized populations, the pair-correlation is computed for each set of coordinates. The baseline or first local minimum of the pair-correlations is then used to determine an appropriate cutoff distance for distance-based clustering. Finally, the molecules from the two sets are classified as colocalized if they lie within the distance determined from the cross-correlation and previously determined cluster information is used to enforce stoichiometry restrictions, improving noise rejection. The cross-correlation and colocalizations steps are the critical steps where using a range search for distance tabulation yields decreased memory requirements compared to an explicit distance matrix.


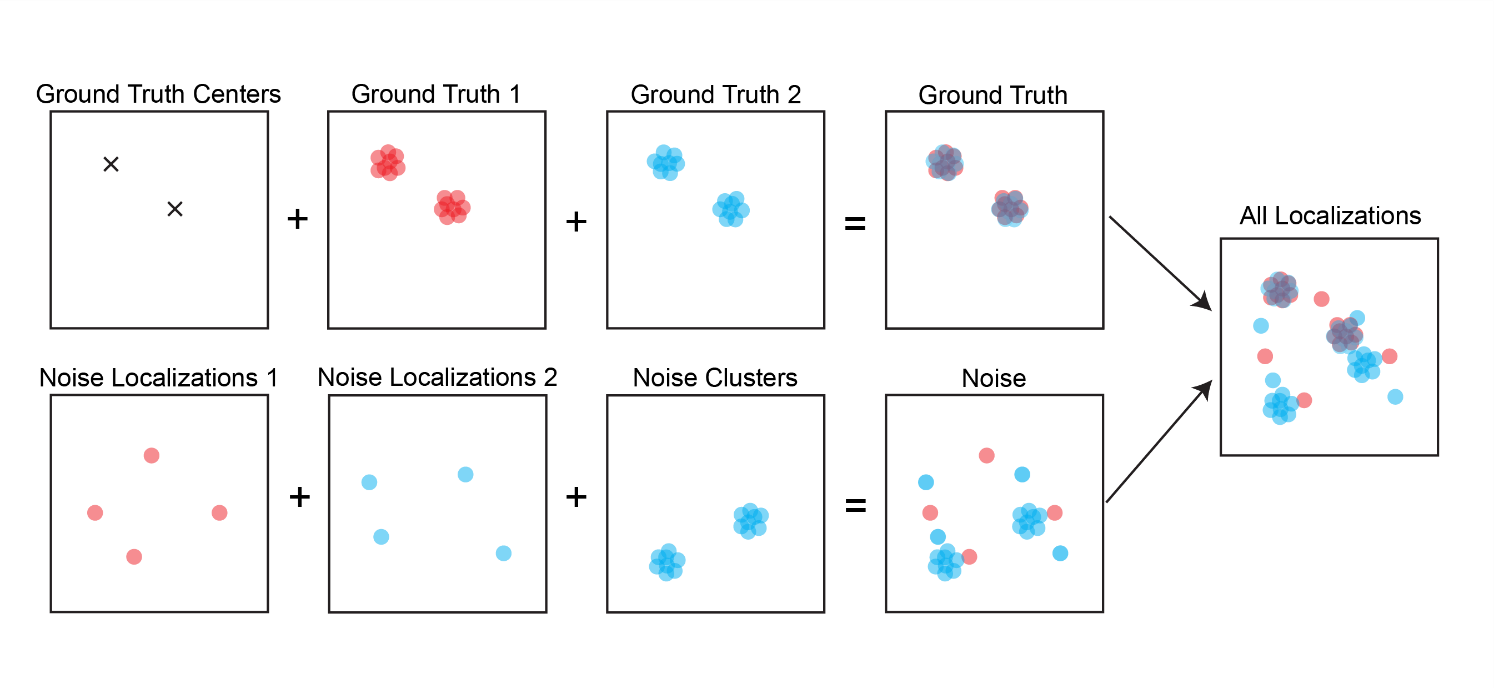


**Supplementary Figure 2: Creation of simulated data**. To construct the simulated data shown and evaluated in Figure 2 and further evaluated in Figure S3, 40 points randomly drawn from a uniform distribution were designated as the centroids of two colocalized populations of ground truth clusters, depicted by an “x.” For the first population (red), coordinates representing single-molecule localizations were distributed about each point from a normal distribution with standard deviation 0.1 ± 0.02 µm. The number of localizations placed around each point was drawn from a Poisson distribution with a mean of 90 localizations. For the second population (blue), the same centroids and standard deviations were used, but the mean number of localizations was 150. The two sets of colocalized clusters combined make up the “ground truth” in the simulation (purple). To simulated noise localizations, 500 points were randomly drawn from a uniform distribution separately into each population. To simulate non-colocalized "noise clusters", 40 points were randomly drawn from a uniform distribution and localizations drawn from a normal distribution with standard deviation 0.1 ± 0.02 µm were distributed around those points. The number of localizations placed around each noise cluster was drawn from a Poisson distribution with a mean of 250 localizations. The 500 noise localizations in population 1, 500 noise localizations in population 2, and 40 noise clusters in population 2 make up the “noise” in the simulation. Altogether, the combined “ground truth” and “noise” comprised the data used in the analysis.

**
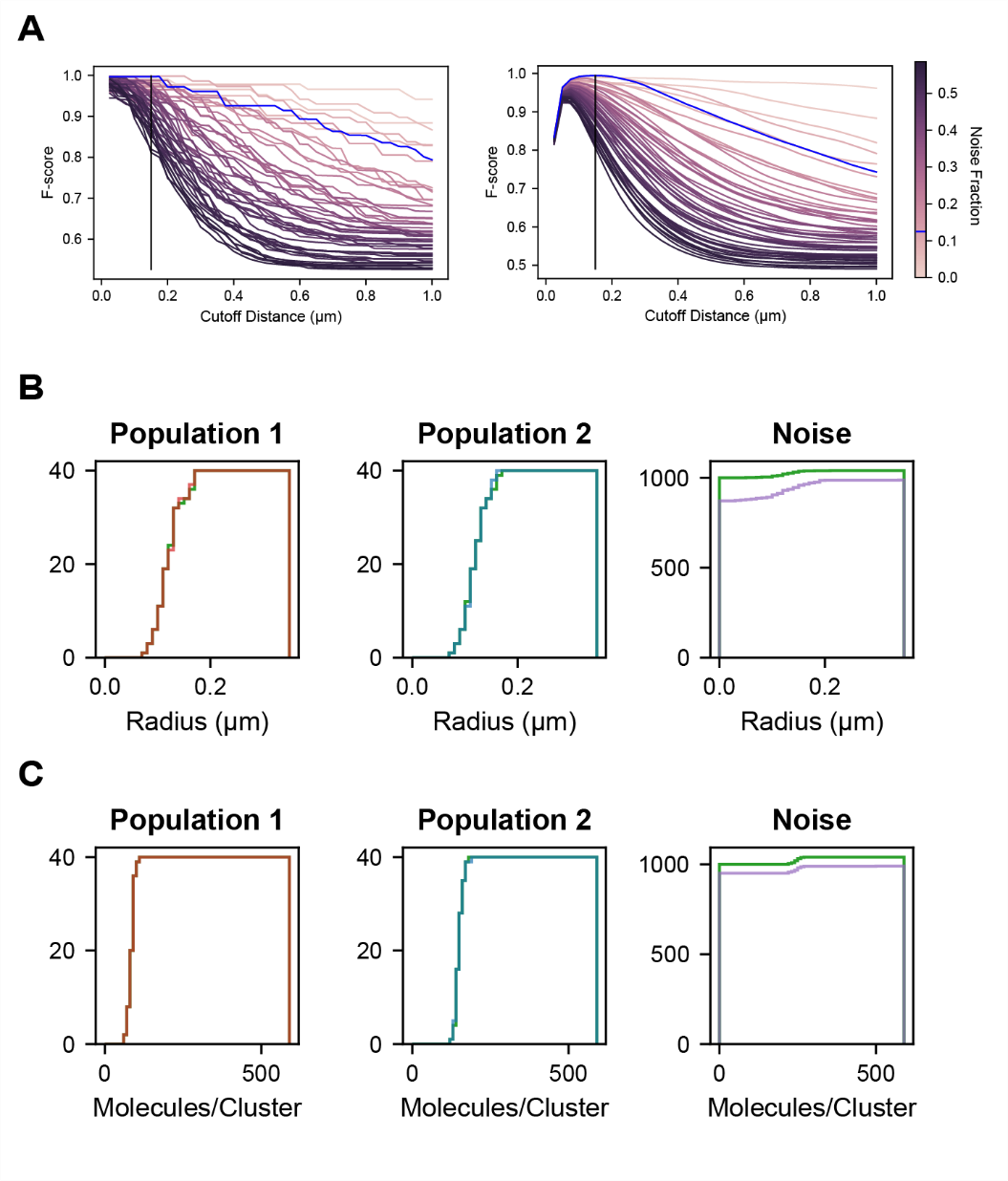
**

**Supplementary Figure 3. Noise variation and recovery of the ground truth** (A) To quantify the recovery of the ground truth clusters as a function of the cutoff distance between localizations, we computed the F-score of the individual localizations in the simulation from Figure 2 with (A, left) and without (A, right) clustering and stoichiometry filtering, varying the number of randomly distributed noise localizations. The same number of noise localizations are added to each population and the noise is expressed as a fraction of the total number of points in population 1 (that only includes colocalized clusters). The blue line corresponds to the same noise points as in the original simulation from Figure 2 (500, noise fraction 0.124). The vertical line represents the mean half maximum cutoff distance calculated from the cross-correlation. The cutoff distance coincides well with the peak of the F-score for the noise level used in the simulation (blue), indicating it is an optimal compromise between obtaining highly colocalized clusters and rejecting clusters that may be in proximity but not completely overlapping. In the case with no clustering and stoichiometry filtering, as the noise is increased, the distance corresponding to the maximum F-score decreases as does the maximum value of the F-score itself, indicating the increased prominence of false negatives. With clustering and stoichiometry filtering ($N\geq2$ for both populations), the F-score is more robust at shorter distances. (B) Cumulative distributions of the radius and (C) molecules/cluster for the ground truth (green) and recovered clusters. From Mann-Whitney U tests on the number and size distributions, the similarity between ground truth and recovered distributions is at least 98% in population 1 and at least 93% in population 2.


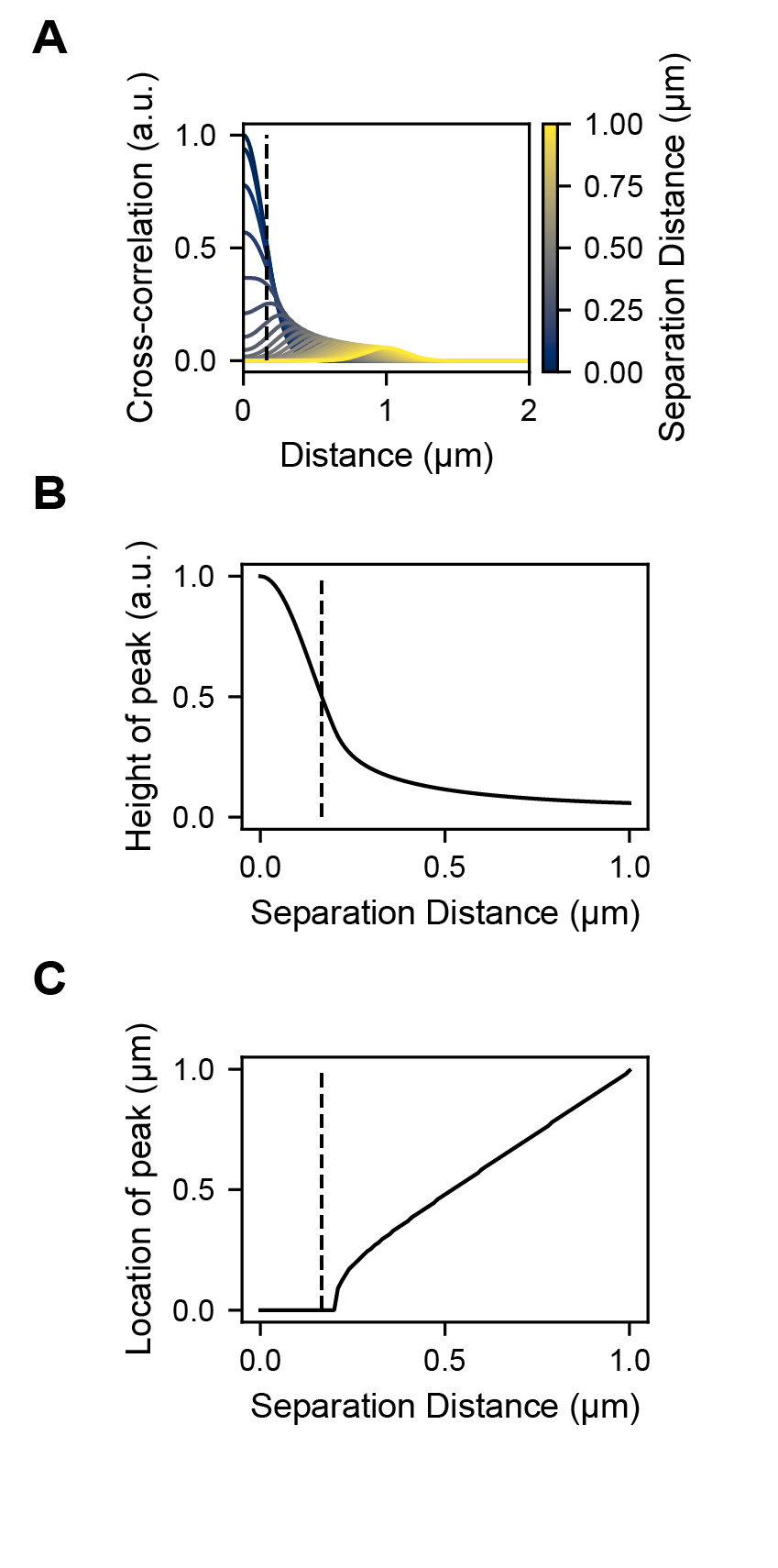


**Supplementary Figure 4: Selection of the cutoff distance for molecule separation.** (A) The cross-correlation function for two Gaussian distributions of standard deviation 0.1 µm and varying separation. The half-maximum of the cross-correlation when the separation is zero is shown as a vertical dashed line. (B) The height of the peak in the cross-correlation as a function of the separation distance between the two clusters. (C) The location of the peak as a function of the separation distance between clusters. Until 0.2 µm, the separation between clusters is not apparent from the cross-correlation, suggesting that the half-maximum is sufficient to associate overlapping Gaussian clusters without associating clusters that are merely proximal to each other.


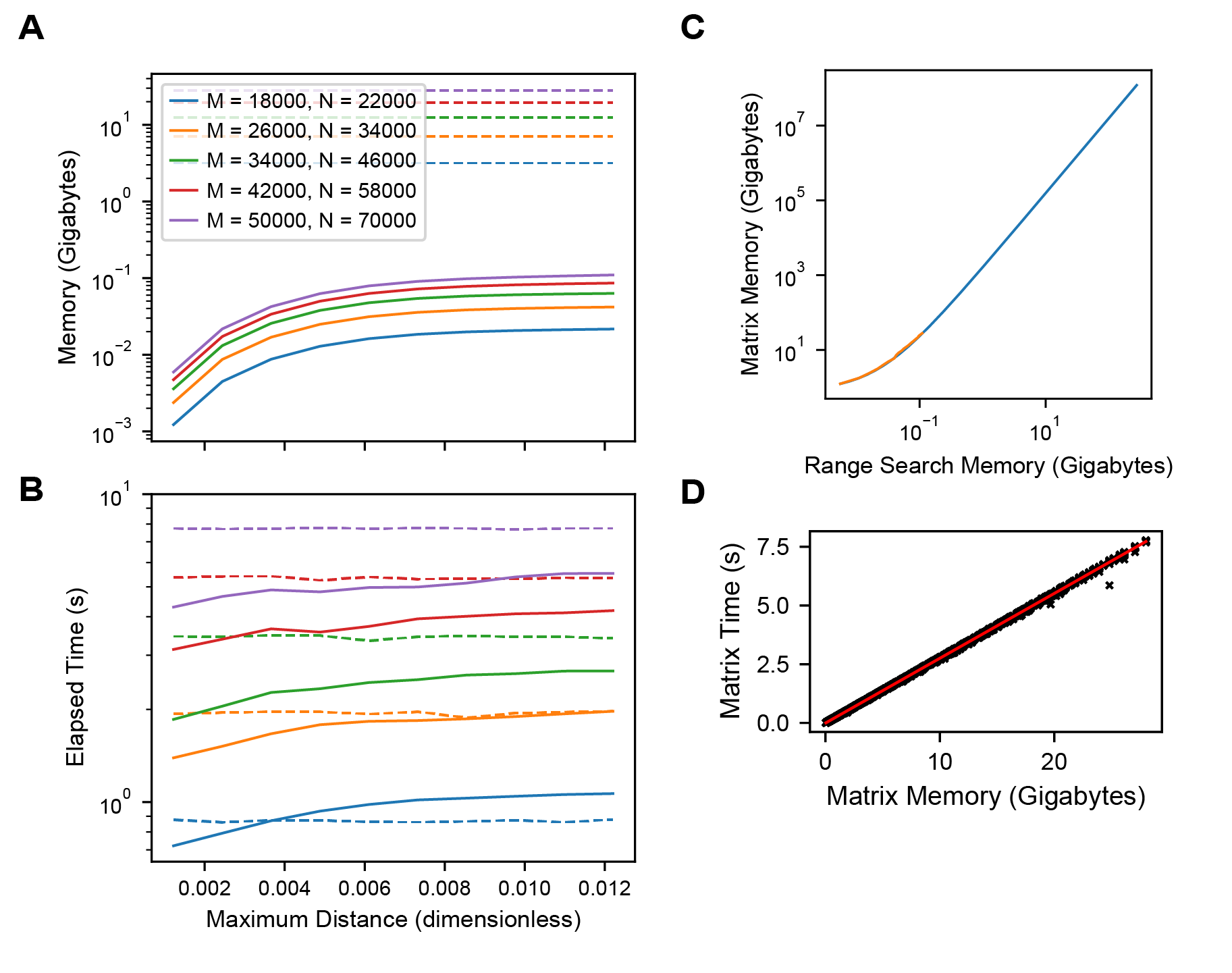


**Supplementary Figure 5: Extreme memory consumption by distance tabulation.** (A) Reduced version of the benchmark simulations described in Figure 3 using data that can be allocated in memory and (B) the corresponding measured time of the distance tabulation. To test the memory and time efficiency of the k-dimensional tree-based cross-correlation compared to full distance matrix approach, we simulated two populations of completely colocalized clusters of stoichiometries of 200 and 300 localizations normally distributed about the centroid with standard deviation 100 nm and 10000 individual noise localizations in each population within a 40.96 µm square field of view. We varied the total number of clusters within the field of view to increase the memory requirements of the cross-correlation. (C) The extrapolation of memory consumption (blue) of the explicit matrix vs. range search for two sets of localizations consisting of clusters and noise, compared to the benchmark data from Figure 2 at a cutoff distance of 0.5 µm (orange). The relation approaches a power-law as the number of localizations is increased by increasing the number of clusters. (D) Linear relation between the memory allocation and time of allocation for the distance matrix for 2200 measurements similar to (A) and (B) with noise localizations ranging from 0 up to 10000. This linear fit of 0.276 s/GB was used to extrapolate the allocation time of matrices in the benchmarks of Figure 3.


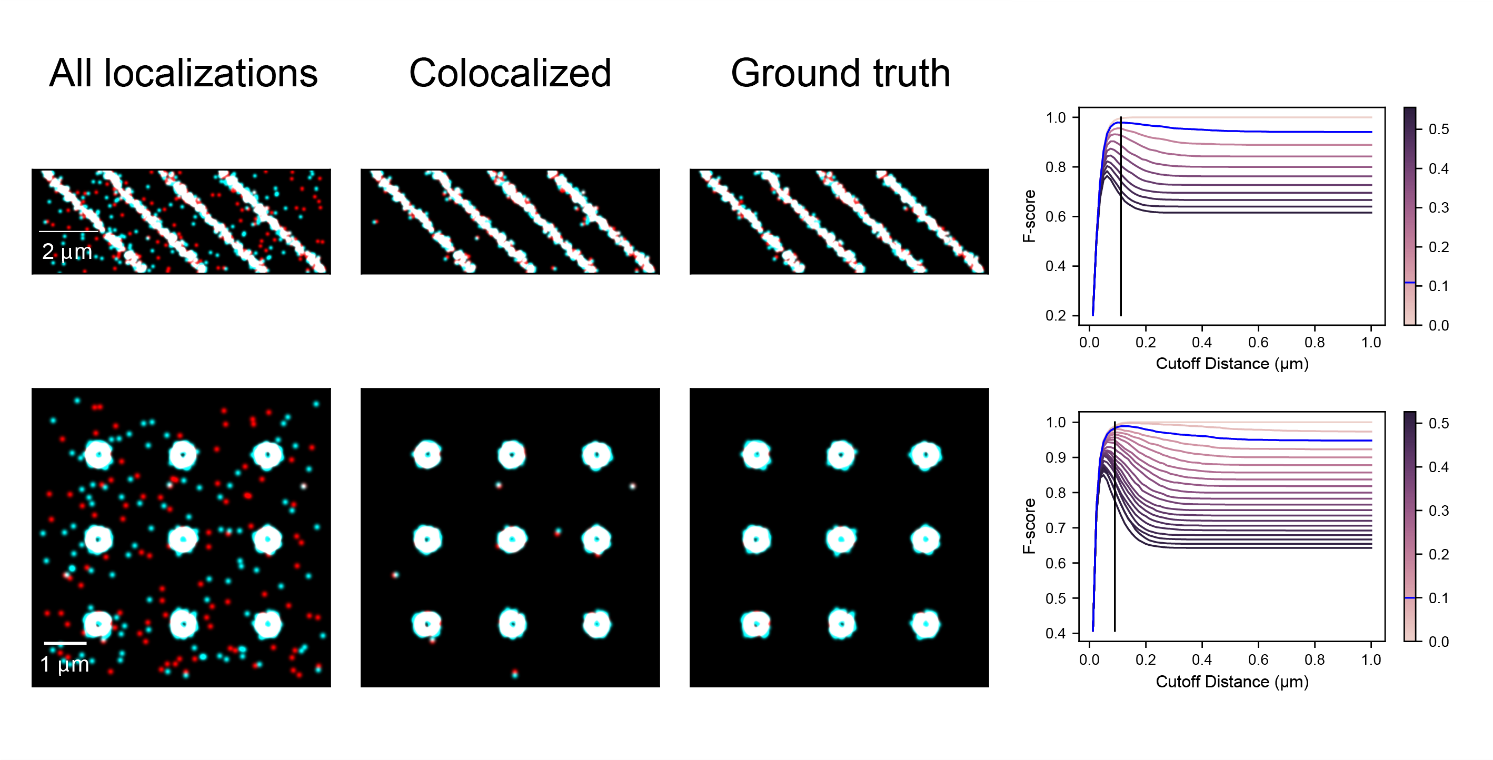


**Supplementary Figure 6: Molecule separation on different geometries.** We tested the molecule separation performance on different geometries. No clustering was performed before colocalizing the two sets of molecules. Top row: all localizations, colocalized localizations, ground truth localizations and F-score evaluation for lines consisting of 200 localizations each. The blue line in the F-score evaluation denotes the case of 100 noise localizations (noise fraction 0.11) shown in the rendering. Bottom row: all localizations, colocalized localizations, ground truth localizations and F-score evaluation for rings consisting of 100 localizations each. The blue line in the F-score evaluation­ denotes the case of 100 noise localizations (noise fraction 0.10) shown in the rendering.
